# Random X chromosome inactivation in patients with Klinefelter syndrome

**DOI:** 10.1186/s40348-020-0093-x

**Published:** 2020-01-24

**Authors:** Kenichi Kinjo, Tomoko Yoshida, Yoshitomo Kobori, Hiroshi Okada, Erina Suzuki, Tsutomu Ogata, Mami Miyado, Maki Fukami

**Affiliations:** 1 Department of Molecular Endocrinology, National Research Institute for Child Health and Development, Tokyo, Japan; 2 Department of Pediatrics, Hamamatsu University School of Medicine, Hamamatsu, Japan; 3 Department of Advanced Pediatric Medicine, Tohoku University School of Medicine, Tokyo, Japan; 4 Department of Urology, Saitama Medical Center, Dokkyo Medical University, Koshigaya, Japan

**Keywords:** Aneuploidy, DNA methylation, Skewed inactivation, Sex chromosome, 47,XXY

## Abstract

**Background:**

X chromosome inactivation (XCI) is an indispensable process in the development of human female embryos. Reportedly, XCI occurs when a blastocyst contains 10–12 embryonic progenitor cells. To date, it remains unclear whether XCI ratios are normally preserved in Klinefelter syndrome (KS) patients with 47,XXY karyotype.

**Methods:**

We examined XCI ratios in 18 KS patients through DNA methylation analysis for the polymorphic trinucleotide locus in the *AR* gene. The results of the KS patients were compared to previous data from healthy young women.

**Results:**

XCI ratios in KS patients followed a normal distribution. Skewed XCI was observed in two patients, one of whom exhibited extremely skewed XCI. The frequencies of skewed and extremely skewed XCI in the KS cohort were comparable to those in healthy women.

**Conclusion:**

This study confirmed the rarity of skewed XCI in KS patients. These results indicate that the presence of a supernumerary X chromosome during the cleavage and early blastocyst stages does not affect the developmental tempo of embryos. Our data deserve further validation.

## Background

In human female embryos, one of the two X chromosomes undergoes X chromosome inactivation (XCI) [1]. The initial step of XCI, which occurs at the early blastocyst stage, is selection of the target X chromosome in each cell [1, 2]. Theoretically, maternally and paternally derived X chromosomes have an equal chance of being inactivated, providing an expected XCI ratio in the body of 50%:50% [1, 2]. However, XCI ratios can be skewed by the intrinsic stochasticity of target selection [3]. In addition, clonal expansion due to growth advantages (or disadvantages) of one X chromosomal allele also results in skewed XCI, although such secondary skewing is observed mostly in elderly women or women with X chromosomal rearrangements/mutations [4, 5]. In 2006, Amos-Landgraf et al. studied 590 healthy female neonates and reported that the XCI ratios of these individuals followed a normal distribution with skewed XCI and extremely skewed XCI accounting for 4.9% and 0.5% of the subjects, respectively [6]. These results indicated that XCI starts when a blastocyst contains 10–12 embryonic progenitor cells. Recently, we showed that the frequency of skewed XCI is increased in individuals with uniparental disomy (UPD) due to monosomy rescue or trisomy rescue/gamete complementation [7]. We proposed that life-saving aneuploid rescue increases the frequency of skewed XCI by reducing the size of embryonic progenitor cell pools at the onset of XCI. However, it remains to be clarified whether the reduced cell pool size in UPD embryos at the XCI onset is simply ascribed to the death of non-rescued cells after aneuploid rescue. Since previous studies have associated embryonic aneuploidy with nuclear abnormalities and decreased developmental potential [8, 9], the reduced cell number of UPD embryos may reflect the impaired development of aneuploid cells during cleavage or early blastocyst stages.

Klinefelter syndrome (KS) is a relatively common disorder in males resulting from sex chromosomal aneuploidy [10]. Patients with KS carry a 47,XXY karyotype and typically exhibit tall stature and gonadal dysfunction [10]. Previous studies have shown that one of the two X chromosomes in KS patients is subjected to XCI [11]. However, it remains uncertain whether these chromosomes undergo random inactivation, because previous studies yielded conflicting results in which the frequency of skewed XCI ranged from 9.1 to 50.0% [11–16]. These inconsistent results possibly result from technical difficulties of XCI analyses or limited number of subjects [17]. If KS is actually associated with XCI skewing, this may suggest that 47,XXY blastomeres have less growth potential than euploid embryos. The present study aimed to clarify the distribution of XCI ratio and the frequencies of skewed and extremely skewed XCI in KS patients.

## Subjects and methods

### Participants

We investigated XCI ratios in 18 children and young adults with KS. These patients visited Dokkyo Medical University because of undermasculinized external genitalia or male infertility. They were diagnosed with KS based on the 47,XXY karyotype. Patients exhibiting a mosaic karyotype were excluded from this study.

### XCI analysis

Genomic DNA samples were obtained from patients’ peripheral leukocytes using the Gentra Puregene blood kit (Qiagen, Valencia, CA, USA). A sample from a healthy male individual was used as a control. The quality of DNA samples was confirmed by gel electrophoresis and the Nanodrop spectrophotometer (Thermo Fisher Scientific, Tokyo, Japan). To determine the XCI ratio, we performed *Hpa*II-mediated DNA methylation analysis for the polymorphic trinucleotide locus in the *AR* gene. The methods were as described previously [18]. The accuracy of the experiments in our laboratory has previously been confirmed by analyzing more than 200 DNA samples from healthy individuals [7]. The XCI ratio was expressed as the ratio of inactive X chromosomes with less CAG repeats to those with more repeats [14].

### Statistical analysis

The distribution of XCI ratios of KS patients was statistically analyzed by employing the Shapiro-Wilk test. In addition, the frequency of skewed XCI (≥ 80%:20% or ≤ 20%:80%) and extremely skewed XCI (≥ 90%:10% or ≤ 10%:90%) in the KS cohort was compared with our previous data obtained from 208 healthy young women [7], by using Fisher’s exact test. Statistical analyses were performed with the IBM SPSS Statistics software (version 24.0).

## Results

### XCI analysis

**Fig. 1:**
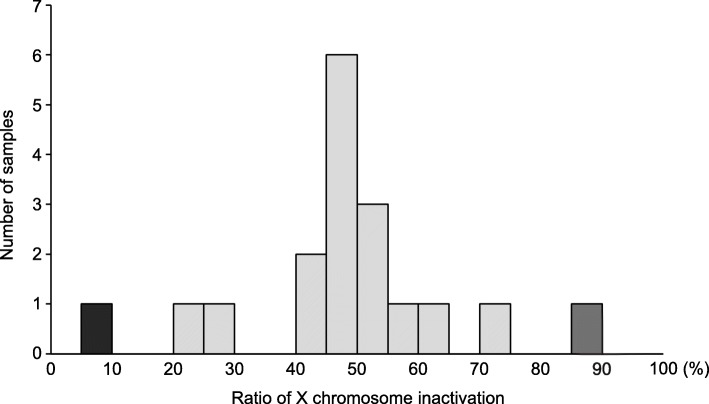
The distribution of X chromosome inactivation (XCI) ratios in 18 patients with Klinefelter syndrome. Inactivation ratios of the shorter (carrying less repeat number) and longer alleles are shown. Skewed XCI (ratios of ≥ 80%:20%, or ≤ 20%:80%) was observed in two of the 18 patients (gray and black bars). Of these, one exhibited extremely skewed XCI (ratios of ≥ 90%:10%, or ≤ 10%:90%) (the black bar)

The results of XCI analysis of the patients and the control individual are shown in Fig. 1 and Additional file 1: Figure S1. The single peak of the male control sample was eliminated after *Hpa*II digestion, confirming a complete enzymatic reaction.

### Statistical analysis

XCI ratios of the 18 patients were normally distributed (mean, 51.6%:48.4%; standard deviation, 18.0%; *p* = 0.270) (Fig. 1). Skewed XCI was observed in two of the 18 patients (11.1%). Of these, one (5.6%) showed extremely skewed XCI. The XCI ratios of the two cases were 13.3%:86.7% and 91.6%:8.4%. The frequencies of skewed XCI and extremely skewed XCI in the KS cohort were comparable to those in healthy young women [7] (11.1% vs. 11.5%, *p* = 0.641 and 5.6% vs. 1.9%, *p* = 0.342, respectively).

## Discussion

We demonstrated that the XCI ratios of KS patients followed a normal distribution. Furthermore, the frequencies of skewed XCI and extremely skewed XCI in KS patients were comparable to previous data obtained from healthy young women [7]. These findings are consistent with previous reports by Zitzmann et al. [15] and Zinn et al. [16], which documented apparently random XCI in KS patients. Considering that excessive X chromosomes in KS patients are equally transmitted from the fathers and mothers [19], the parental origin of X chromosomes appears to have no effect on the XCI target selection. More importantly, the results of this study indicate that, at the timing of XCI onset, embryos with 47,XXY karyotype contain almost equal number of embryonic progenitor cells to that of normal 46,XX embryos.

We further expand this assumption to propose that the presence of an excessive X chromosome in each cell of 47,XXY blastomeres is unlikely to exert a negative effect on cell growth or division. The apparently normal growth of 47,XXY blastomeres may reflect less deleterious effects of sex chromosomal aneuploidy than those of autosomal trisomy [20], because previous studies have linked aneuploidy to the risk of developmental arrest of early stage embryos [8]. Notably, however, our conclusion is based on the observation of live-born individuals. Therefore, we cannot exclude the possibility that 47,XXY embryos more frequently undergo developmental arrest and resultant implantation failure than euploid embryos. Moreover, considering the small number of participants of this study, our data need to be validated in future studies.

In conclusion, this study confirmed the rarity of skewed XCI in KS patients. These results indicate that the presence of a supernumerary X chromosome during the cleavage and early blastocyst stages does not affect the developmental tempo of embryos. Our data deserve further validation.

## Supplementary information

**Additional file 1: Figure S1.** Representative results of X chromosome inactivation analysis. The results of random and skewed X chromosome inactivation (XCI) in the patients, together with the results of a male control, are shown. The elimination of the single peak in the male control sample after *Hpa*II digestion confirms a complete enzymatic reaction.

## Data Availability

The datasets used and analyzed during the current study are included in this published article.

## Abbreviations

KS: Klinefelter syndrome
XCI: X chromosome inactivation
