# Supplementary material for: Random X chromosome inactivation in patients with Klinefelter syndrome
Source: Mol Cell Pediatr. 2020 Jan 24;7:1. doi: 10.1186/s40348-020-0093-x (PMC6979883; doi:10.1186/s40348-020-0093-x)
Supplement: Supplementary file 1 — Additional file 1: Figure S1. Representative results of X chromosome inactivation analysis. The results of random and skewed X chromosome inactivation (XCI) in the patients, together with the results of a male control, are shown. The elimination of the single peak in the male control sample after HpaII digestion confirms a complete enzymatic reaction. [file 40348_2020_93_MOESM1_ESM.pptx]

## Slide 1
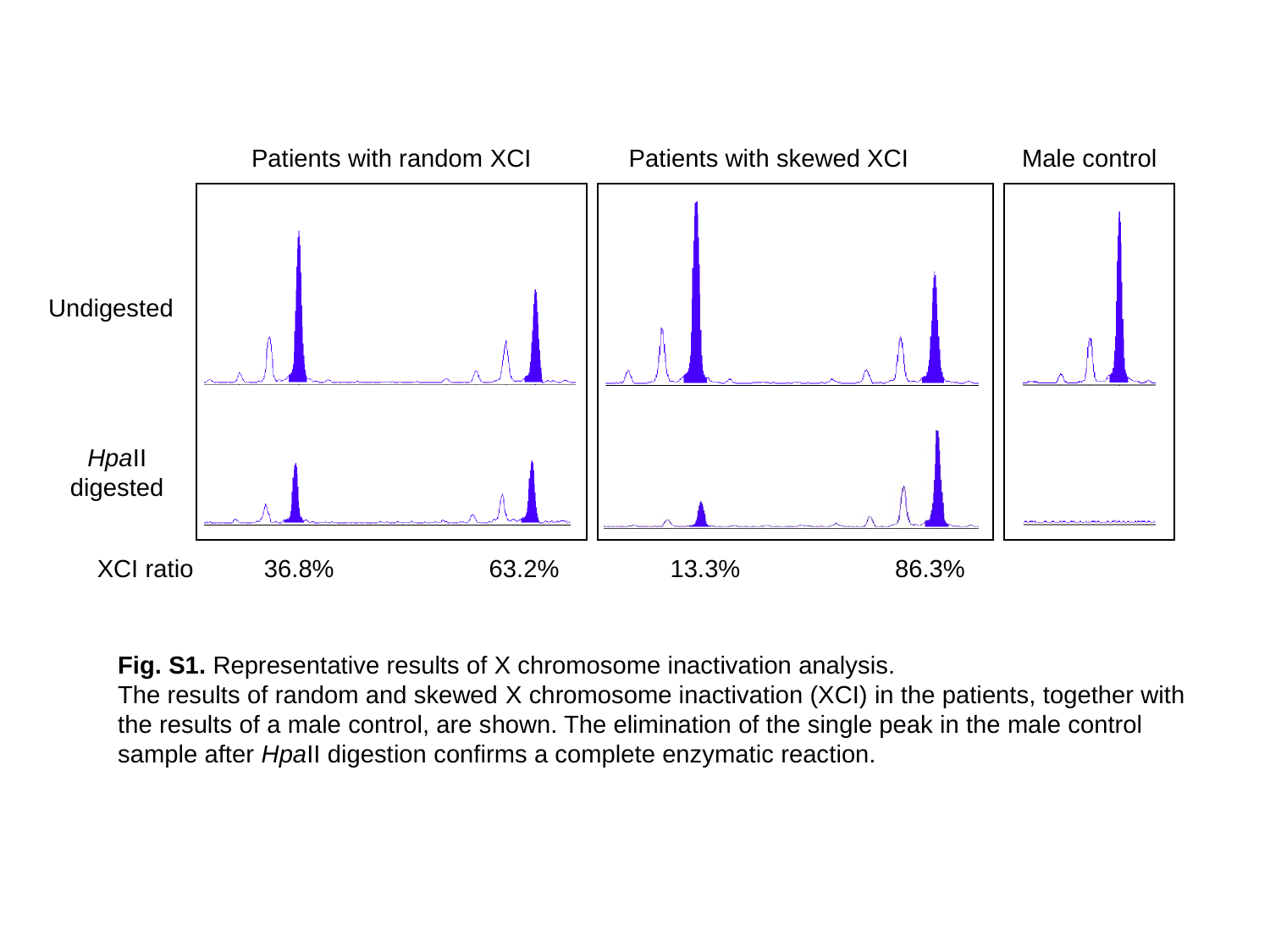

Patients with random XCI
Patients with skewed XCI
Male control
Undigested
HpaII digested
XCI ratio
36.8%
63.2%
13.3%
86.3%
Fig. S1. Representative results of X chromosome inactivation analysis.
The results of random and skewed X chromosome inactivation (XCI) in the patients, together with the results of a male control, are shown. The elimination of the single peak in the male control sample after HpaII digestion confirms a complete enzymatic reaction.
